# Sensitive and Selective Detection of HIV-1 RRE RNA Using Vertical Silicon Nanowire Electrode Array

**DOI:** 10.1186/s11671-016-1504-8

**Published:** 2016-07-22

**Authors:** Jaehyung Lee, Min-Ho Hong, Sanghun Han, Jukwan Na, Ilsoo Kim, Yong-Joon Kwon, Yong-beom Lim, Heon-Jin Choi

**Affiliations:** Department of Materials Science and Engineering, Yonsei University, Seoul, 03722 South Korea; Defense Advanced R&D Center, Agency for Defense Development, Daejeon, 34186 South Korea

**Keywords:** Nanoelectrode, Silicon nanowire, HIV-1 RRE RNA, Biomolecule sensing

## Abstract

In this study, HIV-1 Rev response element (RRE) RNA was detected via an Au-coated vertical silicon nanowire electrode array (VSNEA). The VSNEA was fabricated by combining bottom-up and top-down approaches and then immobilized by artificial peptides for the recognition of HIV-1 RRE. Differential pulse voltammetry (DPV) analysis was used to measure the electrochemical response of the peptide-immobilized VSNEA to the concentration and types of HIV-1 RRE RNA. DPV peaks showed linearity to the concentration of RNA with a detection limit down to 1.513 fM. It also showed the clear different peaks to the mutated HIV-1 RRE RNA. The high sensitivity and selectivity of VSNEA for the detection of HIV-1 RRE RNA may be attributed to the high surface-to-volume ratio and total overlap diffusion mode of ions of the one-dimensional nanowire electrodes.

## Background

During the past decades, many biosensors have been developed for more sensitive and selective detection of target materials under various conditions. Among these, electrochemical sensors have raised interest due to their simplicity, rapid detectability, and low cost together with high sensitivity [1]. Additionally, they provide direct, specific, and real-time detection of target materials [2–4].

One of the critical factors that determines the performance of electrochemical sensors is the electrode where the reactions occur. Conventional macroelectrodes cannot access micro- and nanoenvironments due to their size. The development of nanoscale electrodes allows for the detection of single biomolecules [5] and single cell secretions [6], measurement of local concentration profiles, and in vivo monitoring of neurochemical events [7]. Nanoelectrodes have other advantages regarding diffusion and size. Molecular analytes are consumed at an electrode surface as electrolysis proceeds during an electrochemical redox reaction, making a concentration gradient for fresh analyte to diffuse from the bulk solution. At macroelectrodes, linear diffusion dominates, resulting in limited mass transport. On the other hand, radial diffusion dominates at nanoelectrodes with consequent enhanced rates of analyte mass transport to an electrode. Thus, nanoelectrodes can be used to study faster electrochemical and chemical reactions. In addition, size reduction of each electrode and increase in the total number of electrodes can improve the detection limits and the signal-to-noise (S/N) ratio because the noise level depends on the active area of the individual electrode while the signal depends on the total area of the electrodes [8].

Several nanomaterials have been studied as electrodes including nanoparticles (NPs) [9, 10], nanobelts (NBs) [11, 12], nanorods (NRs) [13], and nanotubes (NTs) [14, 15]. Nanoparticles, commonly used for biosensors, show high sensitivity for detection, but their sensing properties often suffer from degradation as a result of the growth of aggregates among the nanoparticles when operated at high temperature for long times [16]. Moreover, difficulties in controlling the particle size deviation and uniform dispersion hinder the development and characterization of nano-integrated devices.

Nanowires (NWs), which can solve these problems, have advantages as building blocks for electrochemical sensors, such as relatively simple preparation methods allowing large-scale production, superior stability due to high crystallinity, and very large surface-to-volume (S/V) ratio. The latter are mandatory for fast reaction kinetics and high density loading of a target species and catalyst deposition over the surface for the induction or inhibition of specific reactions [16]. Thus, one-dimensional (1D) NWs are potential candidates for future sensors.

In this study, we propose an electrochemical biosensor by combining the NWs with artificial peptides designed to recognize HIV-1 Rev response element (RRE) RNA. Developing device aspects to enhance sensor properties, we also used artificial peptides instead of intact proteins. Artificial peptides are economic, easy to synthesize, and are small in size. Additionally, it is easy to change their properties by modifying the side chains. Using the advantages of both the NWs and artificial peptides, we demonstrate ultra-sensitive and -selective detection of HIV-1 RRE RNA.

## Methods

### Reagents and Materials

Rink amide MBHA resin LL and all Fmoc-protected amino acids were purchased from Novabiochem. Fmoc-Ebes-OH was purchased from Anaspec. N-Methyl-2-pyrrolidone was purchased from Merck. Other solvents were purchased from Sigma-Aldrich. Peptide was synthesized on Rink Amide MBHA resin LL using standard Fmoc protocols via a Tribute™ peptide synthesizer (Protein Technologies, Inc.). The peptides were purified by reverse-phase high-performance liquid chromatography (HPLC, water-acetonitrile with 0.1 % TFA). Concentration was determined spectrophotometrically using a molar extinction coefficient of TAMRA (80,400 M^−1^ cm^−1^) at 547 nm.

### Growth of Silicon Nanowires and Fabrication of Electrodes

The Au-coated vertical silicon nanowire electrode array (VSNEA) was fabricated using the procedures described previously [17, 18] modified as follows. Si (111) substrate was deposited in 0.1 vol.% 3-aminopropyl triethoxysilane (APTES) solution in absolute ethanol. Au catalysts were then coated by immersing the substrate in the colloid solution having Au particles with 250-nm diameter. The substrate was placed in a chemical vapor deposition (CVD) reactor after washing and drying. Silicon nanowires (SiNWs) were vertically grown (with controlled diameter, length, and growth density) on the substrate by flowing SiCl_4_ and H_2_ gas. To fabricate the VSNEA, SiNWs were added a metal layer (Au = 80 nm and Ti = 10 nm) and a SiO_2_ passivation layer onto the surface of the substrate using a sputtering process at a rate of 5 nm/min. Then, the SiO_2_ passivation layer was selectively etched out to expose the Au tips at the top of the NWs by a complementary metal-oxide semiconductor (CMOS) process.

### Peptide Immobilization and RNA Functionalization

The peptide immobilization on VSNEA was carried out by infusing 50 μL of 40 μM peptide in 25 °C environments. The peptide and Au-coated SiNWs were bound by covalent interaction using cysteine (C; this amino acid is expressed in the sequence of peptide in Fig. 4), which has sulfhydryl group, for stability of binding sites. After 12 h for peptide immobilization, RNA was injected to VSNEA using RNase-free water to functionalize with the peptide, and was further incubated for 12 h. Between every immobilization and functionalization step, the VSNEA was washed with distilled water to remove unattached biomolecules.

### Electrochemical Analysis

The cyclic voltammetry (CV) and differential pulse voltammetry (DPV) measurements were performed with 20 mM potassium ferrycianide in 50 mM phosphate-buffered saline (PBS) solution. CV measurements were carried out in a potential range of 0.8 to −0.8 V and the scan rate was 20 mV/s. Sensitivity and selectivity were measured by DPV with a potential range from −0.2 to 0.6 V, pulse amplitude of 50 mV, and pulse width of 10 ms.

## Results and Discussion

### Growth of SiNWs and Fabrication of Electrodes

**Fig. 1:**
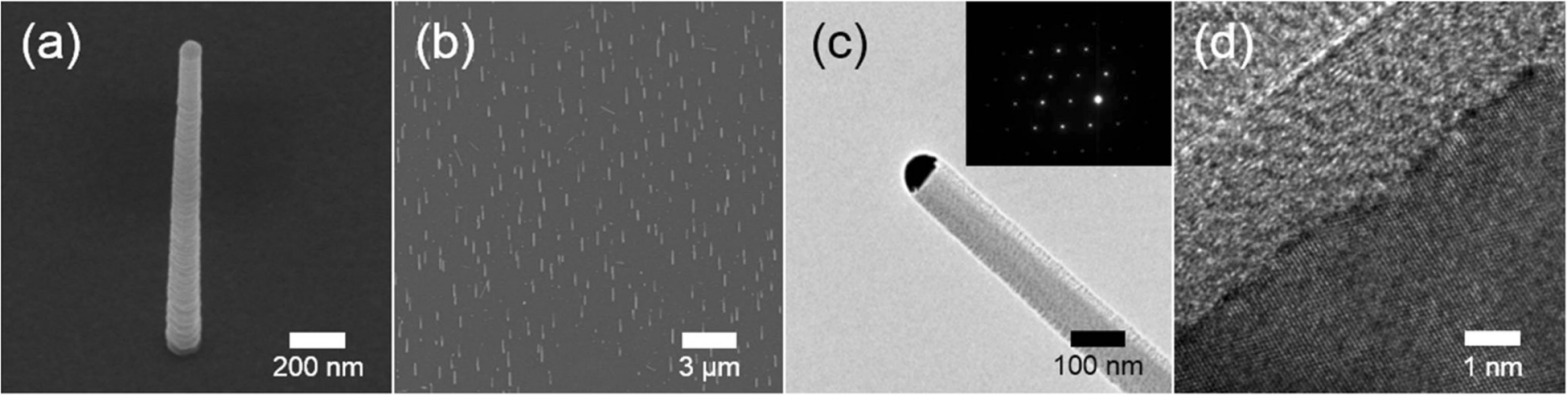
SEM and TEM images of the synthesized SiNWs. **a**, **b** SEM image of vertical SiNWs (tilted by 30°). **c** Typical low-magnitude TEM image and **d** HRTEM image of a SiNW. The *inset* of part **c** shows a SAED pattern of the NW. The *inset* SAED pattern is related with a crystal plane of the NW, and the NW growth direction spots express the [111] crystal planes. It indicates that SiNW has [111] growth direction

Figure 1a, b shows scanning electron microscopy (SEM) images of the SiNWs vertically grown on the substrate. The length of the SiNWs is approximately 3 μm. Figure 1c shows a metal globule at the tip of a NW that indicates the working of the vapor-liquid-solid (VLS) mechanism for the growth of SiNWs. The inset in Fig. 1c shows the selected area electron diffraction (SAED) pattern showing that the SiNWs are single-crystalline and grow along the [111] direction. The diameter of the SiNWs was approximately 200 nm. The single-crystalline nature with a thin native oxide layer and a thickness of about 3 nm is also observed in a high-resolution transmission electron microscopy (HRTEM) image (Fig. 1d).

The diameter and length of the SiNWs could be controlled by the size of Au colloidal particles and growth time. The density of SiNWs on the substrate could also be controlled by the concentration of Au nanoparticles in the colloidal solution. Previous studies have shown that the density and size of NWs that are electrodes of a device can change the properties like sensitivity and selectivity of the NW-based sensors [19].

As mentioned in the introduction, size reduction of each electrode and increase in the total number of electrodes can improve the detection limits and the S/N ratio since the noise level depends on the active area of the individual electrode whereas the signal depends on the total area of the electrodes [8]. In this research, the VSNEA behaves as an electrode with a partially blocked surface operating under total overlap diffusion conditions [20]. Therefore, if we consider voltammetric experiment peak current (the signal, *S*) and capacitive currents (the main component of the noise, *N*) at the VSNEA under a total overlap regime, then we obtain the following equations [19].

1 $$ S={I}_p=2.69\times {10}^5\frac{3}{n^2}{A}_{geom}{v}^{\frac{1}{2}}{D}^{\frac{1}{2}}{C}_b $$

2 $$ N={I}_c={A}_{act}{C}_d $$

$$ \left({A}_{act}= active\kern0.5em  area,{A}_{geom}=\mathrm{total}\kern0.5em \mathrm{geometrica}\kern0.5em \mathrm{area},v=\mathrm{scan}\kern0.5em \mathrm{rate},D=\mathrm{diffusion}\kern0.5em \mathrm{coefficient},C=\mathrm{concentration}\right) $$

Here, the active area, *A*_*act*,_ is the area of the Au-coated, non-passivated surface of nanowires exposed to the medium and the geometric area and *A*_*geom*_, is the total area including the active area and all the passivated surfaces of nanowires and substrates. At conventional electrode, in the same experimental conditions, Eqs. (3) and (4) are given.

3 $$ S={I}_p=2.69\times {10}^5\frac{3}{n^2}{A}_{geom}{v}^{\frac{1}{2}}{D}^{\frac{1}{2}}{C}_b $$

4 $$ N={I}_c={A}_{geom}{C}_d $$

Combining equations from Eqs. (1, 2, 3, and 4) gives Eq. (5).

5 $$ \left(\frac{S}{N}\right)NEA=\left(\frac{S}{N}\right) CONV\times \frac{A_{geom}}{A_{act}} $$

(NEA; nano electrode array, CONV; conventional electrode).

Equation (5) shows that the S/N ratio of the NEA is higher than that at a conventional electrode because *A*_*geom*_/*A*_*act*_ values are generally below 10^−3^ [21]. This means that detection limits of such devices are two to three orders of magnitude better than that of conventional macroelectrodes [22]. The reaction rate can further be enhanced in the nanowire electrodes since a vertically aligned nanowire can work as an “electron antennae” and enhance the electron transfer for the reactions [23, 24].

**Fig. 2:**
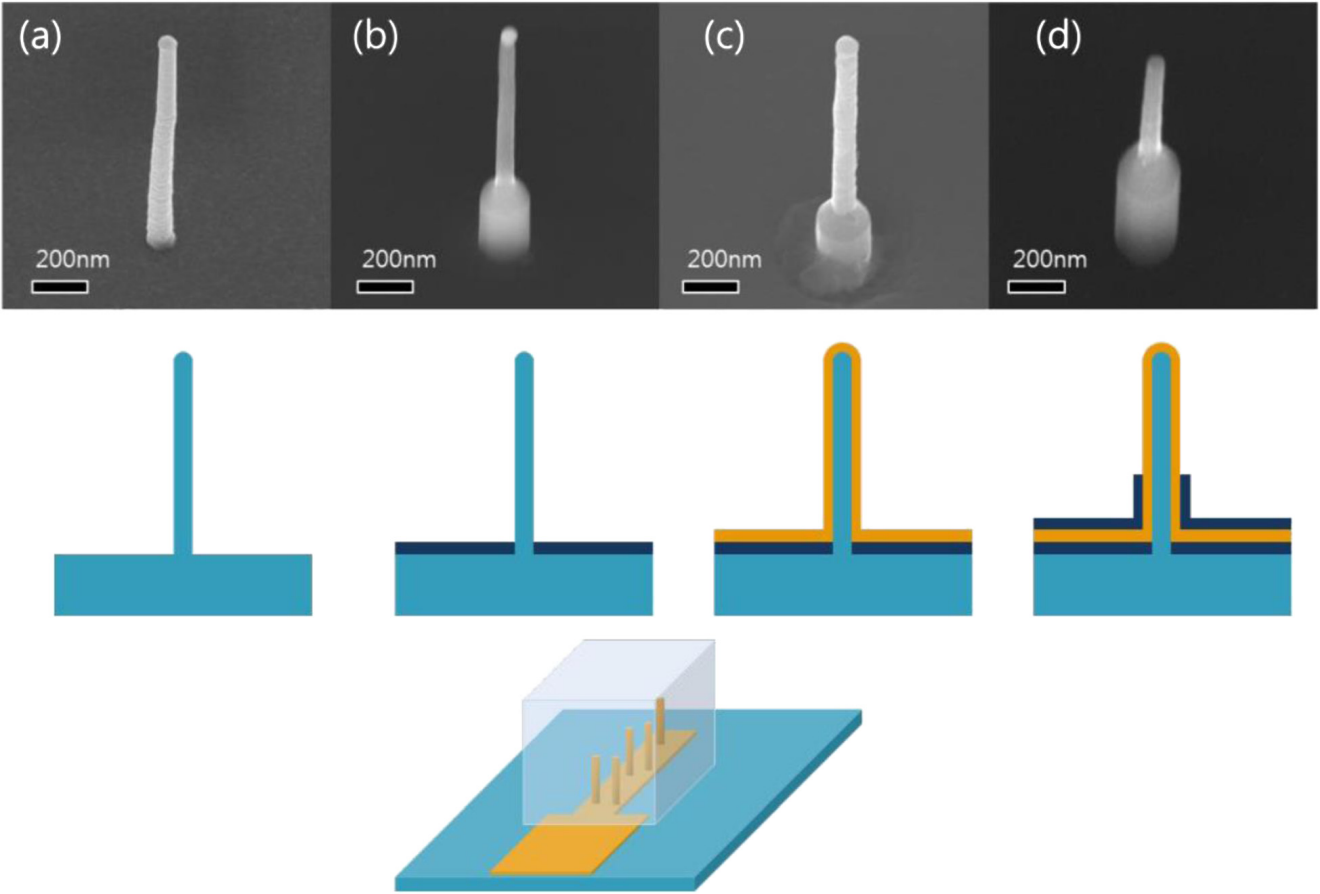
VSNEA fabrication process. **a** Vertical silicon nanowire grown on a (111) Si substrate. **b** First SiO_2_ passivation layer deposition with CVD. **c** Metal-coated SiNWs (Ti 10 nm and Au 80 nm). **d** Second SiO_2_ passivation layer deposition with CVD

Figure 2 shows the scheme of Au-coated VSNEA fabrication using vertical SiNWs. Figure 2a shows the SiNWs grown vertically on the substrate. SiNWs were then covered with SiO_2_ passivation layer through a CVD process and then selectively etched to expose the SiNWs (Fig. 2b). Polymethyl methacrylate resists serving as a SiO_2_ protecting layer was coated on the substrate, and buffered oxide etch was used to selectively etch the SiO_2_ layer on the NWs. Au film was then deposited on the surface of SiNWs to immobilize the peptides (Fig. 2c). Finally, the substrate was coated with a secondary SiO_2_ passivation layer and then selectively etched to expose the Au layer only at the tip of VSNEA (Fig. 2d).

### Immobilization of RNA onto VSNEA

**Fig. 3:**
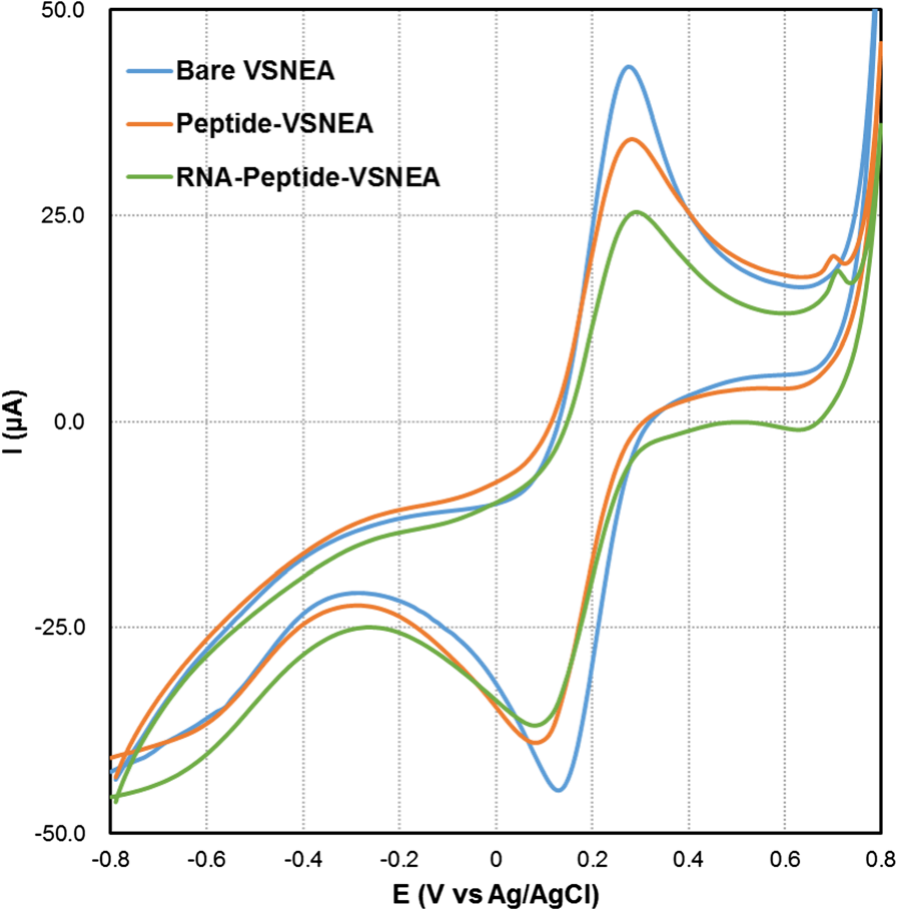
Surface characterization of biosensor using cyclic voltammetry in Fe(CN)_6_^3-/4-^ redox probe

**Fig. 4:**
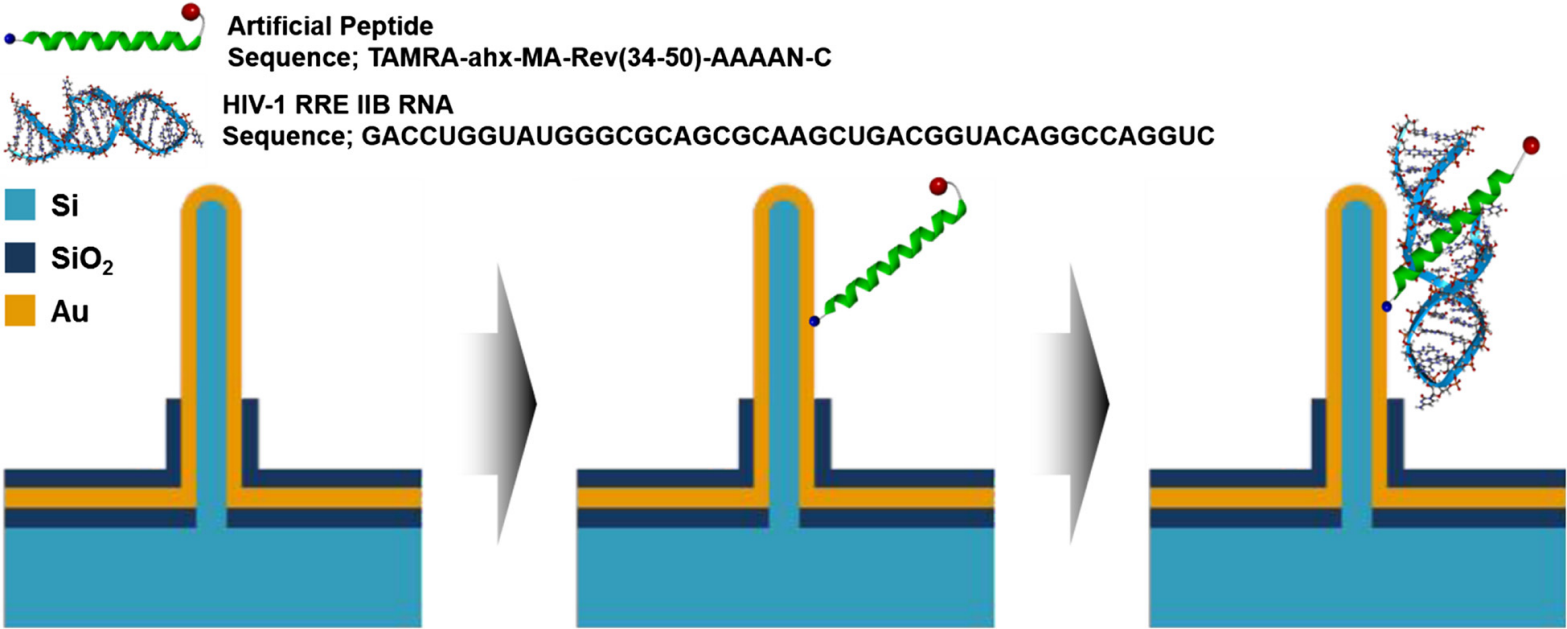
Schematic illustration of principle of immunosensor using VSNEA. As peptides and RNAs are attached to the surface of Au-coated NWs, current path is blocked reducing Fe(CN)_6_^3-/4-^ redox reaction. The peptides were modified in order to be functionalized on the VSNEA (the sequence is shown in *inset*)

The VSNEA in this study was combined with artificial peptides to recognize the HIV-1 RRE. The immobilization of peptides on the surface of VSNEA was investigated by CV measurement. Figure 3 shows CV under bare and immobilized conditions. We use a wide potential scan range to investigate the way of working of this kind of electrodes as well as get the information of electrochemical reactions. The bare VSNEA produces well-defined redox peaks with a cathodic peak potential (*E*_pc_) of 0.28 mV and an anodic peak potential (*E*_pa_) of 0.13 mV in the potential range of 0.8 to −0.8 V with 43.05 μA of anodic peak current (*I*_a_). These peaks are attributed to the redox reaction at the VSNEA surface. The anodic peak current decreases gradually as the peptide (34.32 μA) and the RNA (25.46 μA) were immobilized on the surface of VSNEA. This happens because the peptides and RNA cover the VSNEA surface and interrupt ion transfer with blocking current pathway (Fig. 4). These immobilization and functionalization reduce redox reaction (Fe(CN_6_)^4−^ ↔ Fe(CN_6_)^3−^ + e) inducing peak current decrease. The CV measurements thus confirmed the immobilization of the peptides on the surface of the VSNEA.

### Detection of Different Concentrations of RNA

**Fig. 5:**
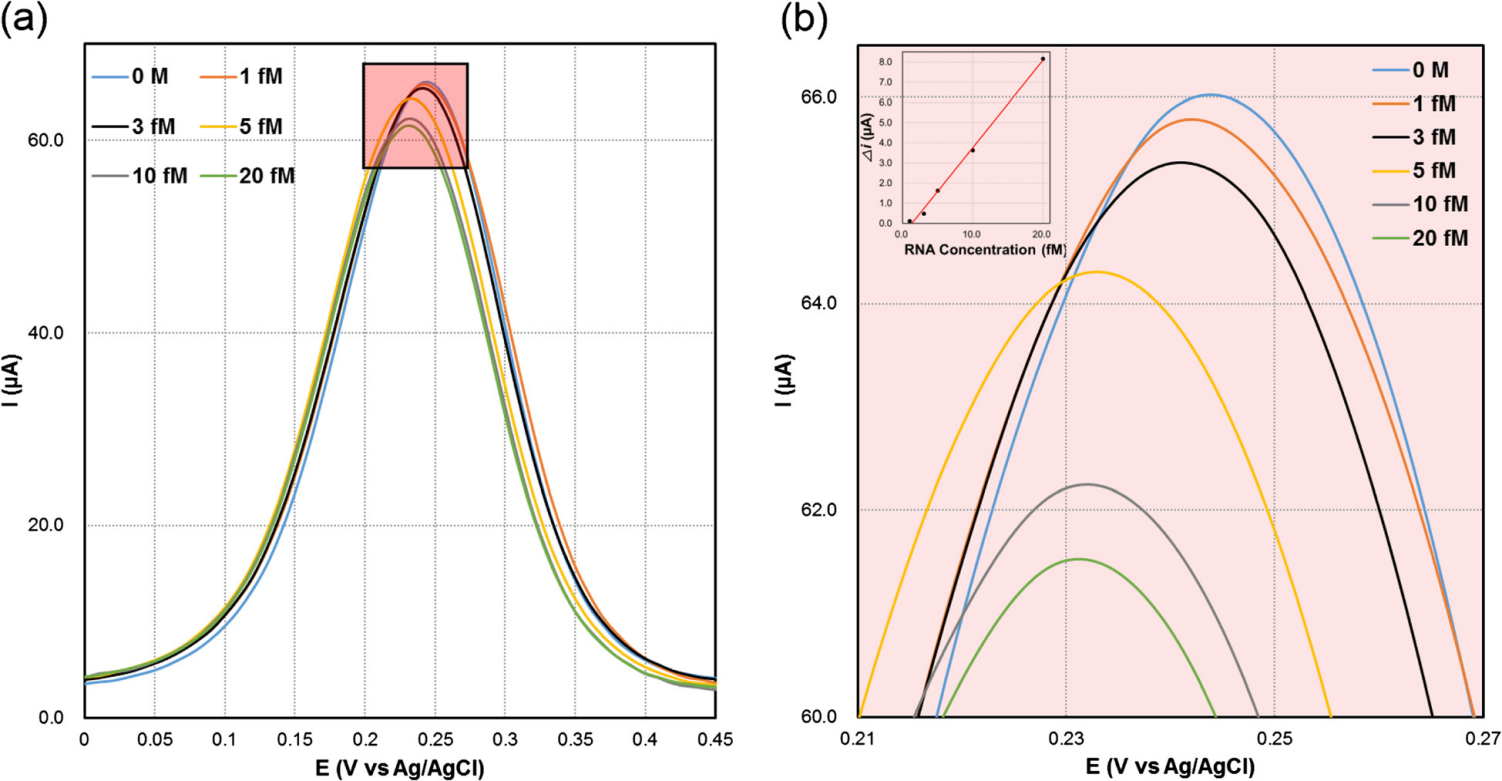
(**a**) Differential pulse voltammograms obtained at HIV-1 RRE RNA-coated VSNEA at different RNA concentrations (0, 1, 3, 5, 10, and 20 fM). The inset shows linear regression obtained from difference between the DPV signals of RNA functionalized VSNEA in the presence and absence of target. (**b**) DPV results at V vs Ag/AgCl in the range 0.21-0.27

The efficiency of VSNEA as a biosensor is explored by detection of RRE IIB RNA. The detection was carried out via DPV, which is a highly sensitive analytical method used for the quantitative determination of analytes. Figure 5 shows DPV signals of RNA functionalized VSNEA which exhibit (a) 65.83, (b) 65.71, (c) 65.34, (d) 64.20, (e) 62.19, and (f) 61.49 μA as the concentration of RRE IIB RNA changes from 1 to 20 fM. This clearly shows that the peak current was proportional to the RNA concentration.

The inset of Fig. 5 depicts the linear correlation of the differences in peak currents, [*I*p(c) = *I*p (c = 0) – *I*p (c)] with respect to the target RNA concentration. The regression equation for the peak current (*I*), and the concentration of the target RNA (c), shows a correlation coefficient (*R*^2^) of 0.99557 with standard deviation of ± 2.310^−7^ fM. Detection limit was calculated to be 1.513 fM. Recent HIV-1 biosensors using nanomaterials such as nanopore [25], nanoparticle [26], and graphene composite film [27] showed detection limits from tens of fM to nM. Compared to these, the detection limits of VSNEA in this study show superior detectability. This is due to the nanosize and unique shape of nanowire as an electrode, which cause enhanced mass transport and fast electron-transfer kinetics [28, 29]. In addition, total overlap diffusion with proper electrode structure, size, and distance between the electrode elements make it possible to achieve very high sensitivity with fM detection limit.

### Detection of Mutated RNA

The working of VSNEA as a biosensor is also explored by the detection of different types of RRE IIB. Three different types [RRE IIB RNA (complementary), RRE IIB mutant and T7 primer DNA] were evaluated by DPV measurement. The sequences used were as follows:

RRE IIB RNA sequence:

GACCUGGUAU**G**GGCGCAGCGCAAGCUGACGGUA**C**AGGCCAGGUC

RRE IIB mutant sequence:

GACCUGGUAU**C**GGCGCAGCGCAAGCUGACGGUA**G**AGGCCAGGUC

T7 primer DNA sequence: TAATACGACTCACTATAGGAG

**Fig. 6:**
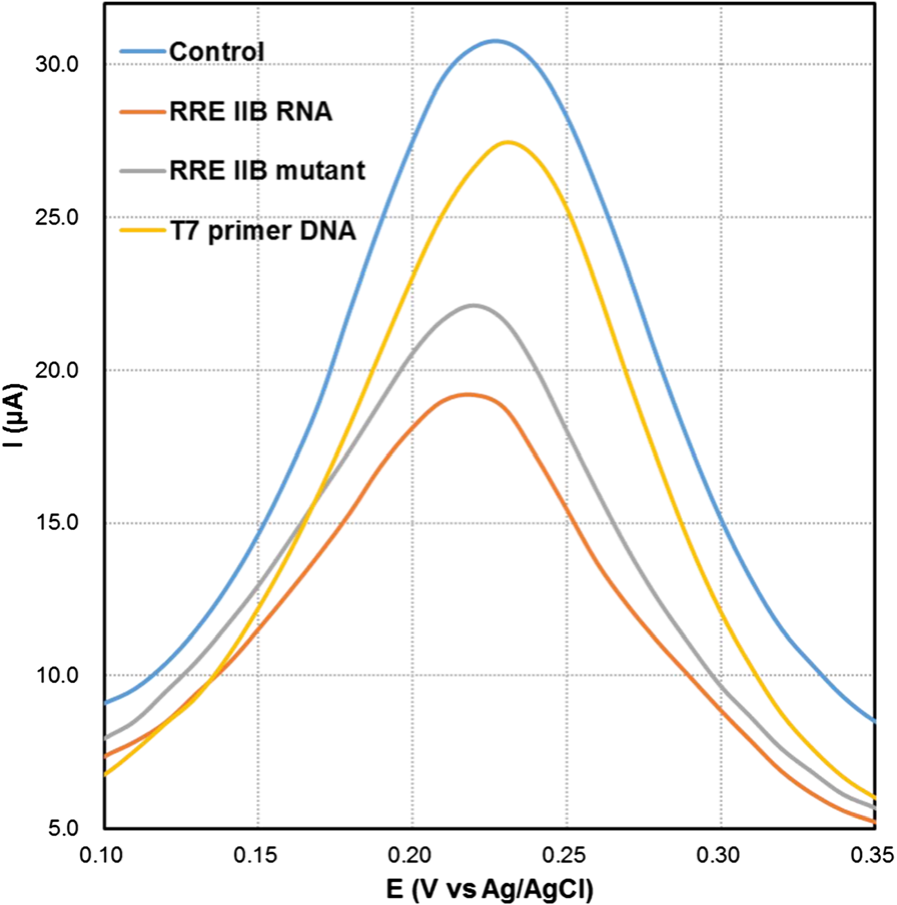
Differential pulse voltammograms of Fe(CN)_6_^3-/4-^ at blank (peptide-coated), RNA-coated VSNEA, RNA mutant-coated VSNEA, and DNA-coated VSNEA

Figure 6 shows DPV responses for the mutated RNA. The bare RRE IIB RNA shows the highest DPV response followed by the RRE IIB mutant and the T7 primer DNA. This is because the Rev peptide specifically binds to RRE IIB wild type and the C46-G74 mutation reduces affinity of the Rev peptide. The well-structured RRE RNA provides space for Rev proteins to bind specifically via interactions such as hydrogen bonds. Base-pair mutations of RNA result in changes of the binding space. DNA provides a completely different space compared with RRE RNA, and there is only an electrostatic interaction between the negatively charged DNA and the positively charged peptide. Therefore, it is harder for Rev proteins to bind DNA or mutant RRE than RRE RNA and these differences of binding interaction induced a DPV peak current change between the blank (30.72 μA) and the T7 primer DNA (27.45 μA), RRE IIB mutant (22.11 μA), and RRE IIB RNA (19.18 μA). Compared to RRE IIB RNA, these two types showed noticeable difference and demonstrate that the electrochemical biosensor based on VSNEA is effective in signaling the presence of a complementary target and discriminating targets from a single base-pair substitution mutation.

## Conclusions

Sensitive and selective detection of HIV-1 RNA by using VSNEA combined with artificial peptides was demonstrated. The advantages of vertical, 1D nanowire electrodes such as high surface area to volume ratio and total overlap diffusion of ions make it possible to detect RNA concentrations of up to 1.513 fM and distinguish between wild-type RNA and mutant RNA. Thus, vertical nanowire sensors may be used as sensitive and selective biosensor platforms in many applications.
